# International normalized ratio measurement during perioperative anticoagulation bridging with low-molecular-weight heparin in patients undergoing heart valve replacement surgery

**DOI:** 10.1016/j.rpth.2024.102616

**Published:** 2024-11-05

**Authors:** Liza Rijvers, Sanna R. Rijpma, Herbert B. van Wetten, Yvonne M.C. Henskens, An K. Stroobants

**Affiliations:** 1 Eurofins Gelre, Eurofins Clinical Diagnostics, Gelre ziekenhuis, Apeldoorn, the Netherlands; 2 Laboratory of Hematology, Department of Laboratory Medicine, Radboud UMC, Nijmegen, the Netherlands; 3 Department of Cardiothoracic Surgery, Radboud UMC, Nijmegen, the Netherlands; 4 Central Diagnostic Laboratory, Maastricht UMC, Maastricht, the Netherlands; 5 Radboud Laboratory for Diagnostics, Department of Laboratory Medicine, Radboud UMC, Nijmegen, the Netherlands

**Keywords:** anticoagulation, heparin, international normalized ratio, low-molecular-weight heparin, vitamin K

## Abstract

**Background:**

Surgical procedures in anticoagulated patients require specific attention due to increased bleeding risk. Preoperative anticoagulation interruption in high-risk patients is often necessary. Bridging anticoagulation with low-molecular-weight heparin (LMWH) minimizes thromboembolic risk, but its effect on international normalized ratio (INR) measurement is not well established, necessitating careful monitoring and individual assessment.

**Objectives:**

To investigate the effect of heparin bridging on INR measurements in anticoagulated patients on vitamin K antagonist (VKA) and in *in vitro* spiking experiments.

**Methods:**

Thirty-eight anticoagulated patients on VKA undergoing valve replacement surgery were studied using 2 plasma-based INR assays and 1 whole blood point-of-care INR method at multiple time points after postoperatively resuming VKA. In addition, INR levels in pooled plasma of both normal and VKA-treated individuals were compared, with 7 spiked concentrations of LMWH or unfractionated heparin (UFH) in 4 INR assays.

**Results:**

In LMWH-bridged anticoagulated patients, the INR results obtained with HemosIL RecombiPlasTin and point-of-care Coaguchek were significantly higher than those obtained with STA Hepato Prest within 3 days after restart of VKA. After spiking LMWH or UFH in various concentrations into pooled plasma, only the STA Hepato Prest assay showed no interference in INR measurement within the therapeutic range (1.0-2.0 international units/mL) in both VKA and normal plasma. All other assays showed substantial interference, with the Thromborel S assay being the most heparin-sensitive assay.

**Conclusion:**

Differences between INR methods are seen within 72 hours after restarting VKA in postoperative patients who receive LMWH bridging. *In vitro* experiments using LMWH and UFH show the interference of heparin in multiple INR methods, even with concentrations below the suppliers’ stated heparin interference limits.

## Essentials

• Bridging with heparin reduces thromboembolic risk but might impact blood clotting tests (international normalized ratio [INR]).
• The effect of heparin bridging on INR in patients on vitamin K antagonists was studied.
• Heparin bridging affects INR results in patients undergoing heart valve surgery.
• Heparin affects INR results, even at levels below the suppliers’ stated interference limits.

## 1 Introduction

Surgical procedures in patients receiving anticoagulation therapy require careful management due to the dual risks of thromboembolic events and bleeding during surgery [1]. Preoperative cessation of anticoagulation is often imperative, especially for high-risk surgeries such as valvular replacement requiring cardiopulmonary bypass [1,2]. In addition to surgical injuries that cause hemostatic activation, cardiopulmonary bypass causes coagulation changes due to factors such as hemodilution, hypothermia, and thrombocytopenia.

During the interruption period, low-molecular-weight heparins (LMWHs) are utilized to minimize the risk of thromboembolic complications, a practice known as “bridging anticoagulation,” but debates on its necessity or the optimal dosing are ongoing [2,3]. LMWHs are preferred due to their predictable dose response, subcutaneous administration, short half-life, and minimal need for routine laboratory monitoring [[4], [5], [6], [7]].For patients on vitamin K antagonists (VKAs), preoperative management involves discontinuing the medication several days prior to surgery to allow for the reduction and re-establishment of its anticoagulant effect.

The timing of VKA discontinuation varies by specific VKA, with acenocoumarol requiring a 3-day interruption and phenprocoumon a 5-day interruption, according to guidelines from Dutch, American (American College of Chest Physicians), and European (European Society of Cardiology) sources. Bridging with LMWH starts when the international normalized ratio (INR) is <2.0, with the last dose administered 24 hours before surgery. INR is closely monitored to ensure the target level is reached before surgery. Postoperatively, LMWH is given 12 to 24 hours after surgery, and VKAs are resumed 24 to 48 hours later. Bridging anticoagulation is stopped after 2 consecutive INR measurements above target values [6,8,9].

INR is calculated using a prothrombin ratio and a correction factor (International Sensitivity Index), assuming international comparability across laboratories [10]. However, INR accuracy can be affected by heparin, even though the reagents contain varying amounts of heparin neutralizers [[11], [12], [13]]. This study investigated the impact of LMWH bridging on INR measurement accuracy in anticoagulated patients undergoing valve replacement surgery. INR measurements were compared using different assays at various postoperative time points, and INR levels were assessed in plasma samples spiked with LMWH or unfractionated heparin (UFH) across 4 assays. The goal was to optimize anticoagulation therapy in postoperative settings by understanding the effect of heparin bridging on INR measurements.

## 2 Methods

### 2.1 INR in postoperative patients on heparin and VKA combination

From 38 patients undergoing valve replacement surgery who were anticoagulated with LMWH, citrated plasma (0.109 mol/L) was collected for INR measurement after restarting acenocoumarol or fenprocoumon postoperatively. The patients received LMWH bridging agents (dalteparin [Fragmin]) until the INR was within 2 times the patients’ therapeutic range, following the Dutch protocol [8]. Although patients sometimes received UFH during surgery, due to its short half-life (1-2 hours), it was no longer detected in the bloodstream on postoperative day 3. The study was performed in accordance with the Declaration of Helsinki, and participants gave general consent for further use of body material. INR was measured at different time points, ranging from 15 to 168 hours (days 1-7) after the first dose of VKAs. Data were separated into groups by days (1-3 days and 4-7 days) after restarting VKA. Two different methods and reagents were used: ACL TOP 350 with RecombiPlasTin reagent (Werfen) and STA R Max with Stago Hepato Prest reagent (Stago). In addition, in 22 of the 38 patients, INR was measured directly with point-of-care testing using Coaguchek Pro II (Roche Diagnostics).

### 2.2 Interference testing (*in vitro*)

The *in vitro* sensitivity to LMWH or UFH of 4 different INR assays was determined: ACL TOP 350 analyzer with HemosIL RecombiPlasTin reagent (both Instrumentation Laboratory), STA R Max analyzer with the STA Hepato Prest reagent (both Stago), CS-2500 analyzer with both Dade Innovin and Thromborel S reagents (all Siemens). Eight different concentrations of LMWH (dalteparin 12,500 International Units per milliliter [IU/mL]) or UFH; Heparin LEO, 5000 IU/mL) were used: 0, 0.5, 0.75, 1.0, 1.5, 2.0, 2.5, and 5.0 IU/mL. Each concentration was spiked into citrated plasma from a pool of nonanticoagulated patients (n = 48) or a pool of patients who had been anticoagulated with vitamin K inhibitors (n = 48). Concentrations of spiked LMWH or UFH were confirmed using anti-Xa measurement with an ACL TOP analyzer using HemosIL Liquid Anti-Xa (Instrumentation Laboratory). To neutralize both heparin types that were present in the spiked samples, heparinase (Dade HepZyme, Siemens) was added in the manufacturer’s recommended concentration (>125 IU heparinase/mL plasma). This concentration can neutralize up to 2 IU heparin/mL plasma, which was confirmed by anti-Xa measurement.

### 2.3 Statistical analyses

Statistical analyses were performed using Graphpad Prism 9 and included the paired *t*-test, Wilcoxon test, Mann–Whitney U-test, Welch’s *t*-test, and 2-way anova. *P* values < .05 were considered statistically significant. All data sets were tested for normal distribution with the D’Agostino and Pearson test. If data sets were not normally distributed, nonparametric tests were used.

## 3 Results and Discussion

**Figure 1:**
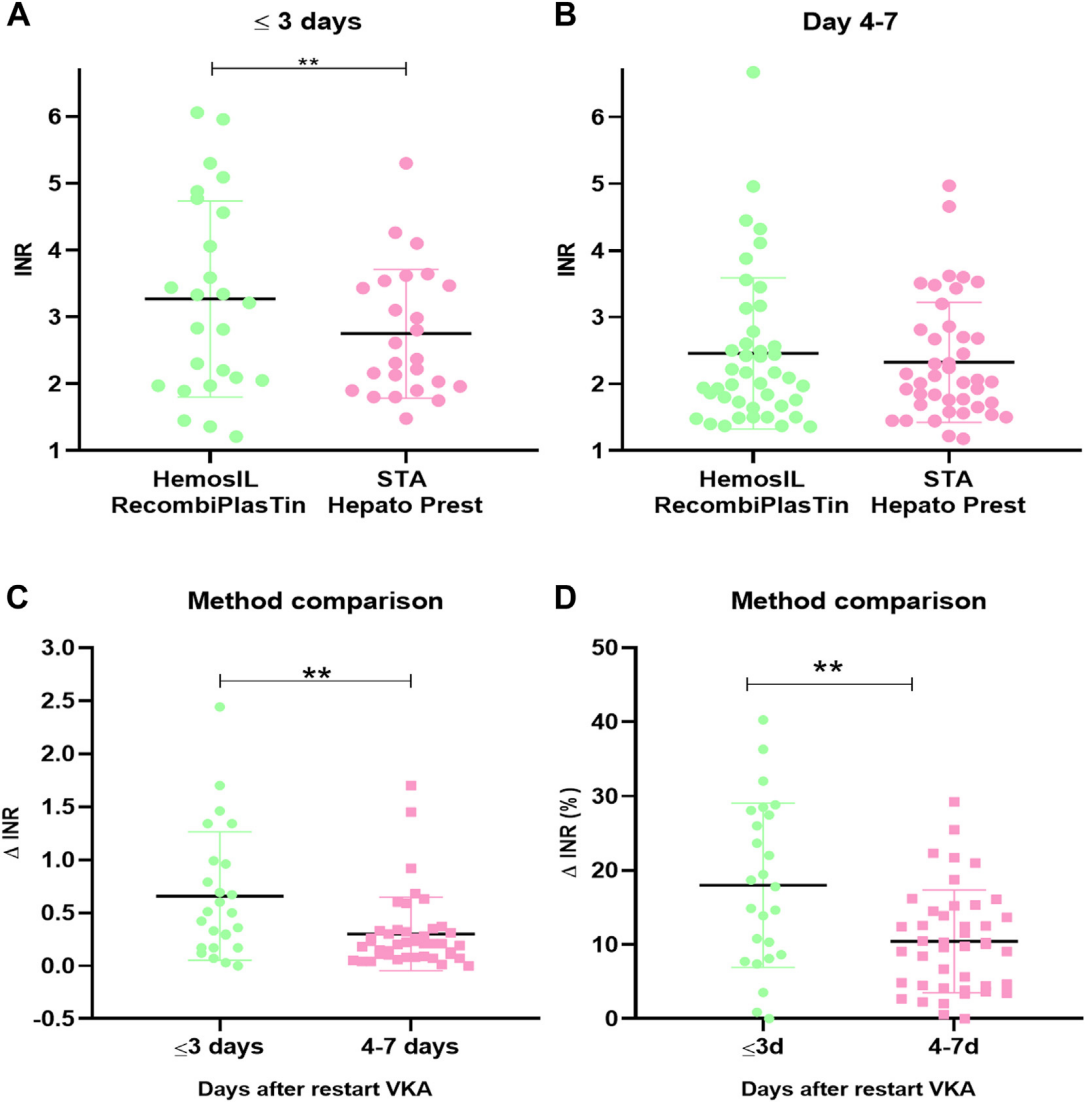
INR measurement in postoperative anticoagulated patients who received heparin bridging due to valve replacement surgery. INR measurement with HemosIL RecombiPlasTin and STA Hepato Prest within the first 3 days (A) or between days 4 to 7 (B) after restarting VKA in patients who underwent valve replacement surgery with anticoagulants bridging with LMWH or UFH. In (C) and (D), the INR differences between the HemosIL RecombiPlasTin and STA Hepato Prest assays were calculated as absolute (C) of relative (D) difference (Δ INR). Statistical differences were tested with a paired *t*-test (A), Wilcoxon test (B), Mann–Whitney U-test (C) and Welch’s *t*-test (D). ∗∗*P* < .01. INR, international normalized ratio; LMWH, low-molecular-weight heparin; UFH, unfractionated heparin; VKA, vitamin K antagonist.

This study examined the effect of LMWH bridging on INR measurements taken ≤72 hours (≤3 days) and >73 hours (4-7 days) after restarting VKA therapy. Two INR methods were used: HemosIL RecombiPlasTin and STA Hepato Prest. Within the first 3 days, INR values obtained with HemosIL RecombiPlasTin were significantly higher than those obtained with STA Hepato Prest (Figure 1A; Supplementary Figure 1A). From day 4 onward, no significant difference was observed between the assays (Figure 1B). The mean differences in INR values (Figure 1C) and percentages (Figure 1D) were significant between the 2 categories. Additionally, point-of-care Coaguchek INR was measured in 22 of 38 patients and showed a significant increase in the first 3 days compared to STA Hepato Prest but not from day 4 onward (Supplementary Figure 1B). Coaguchek INR values were not significantly different from those measured with HemosIL RecombiPlasTin in both time categories (Supplementary Figure 1C).

From these results, we conclude that heparin interference in different INR measurements is minimal from day 4 after restarting VKA, assuming standard LMWH breakdown. When VKA therapy is resumed, the variation in concentrations of active coagulation factors may explain therapeutic INR levels 72 hours after restart but not the INR bias between different assays. LMWH is administered until a normal INR is measured for >24 hours. Assay methods vary, causing INR differences even without LMWH. Additionally, LMWH’s half-life depends on kidney function. Therefore, in the next experiment, the *in vitro* effect of the presence of LMWH or UFH was studied in plasma from VKA-treated patients as well as in normal plasma from healthy volunteers.

**Figure 2:**
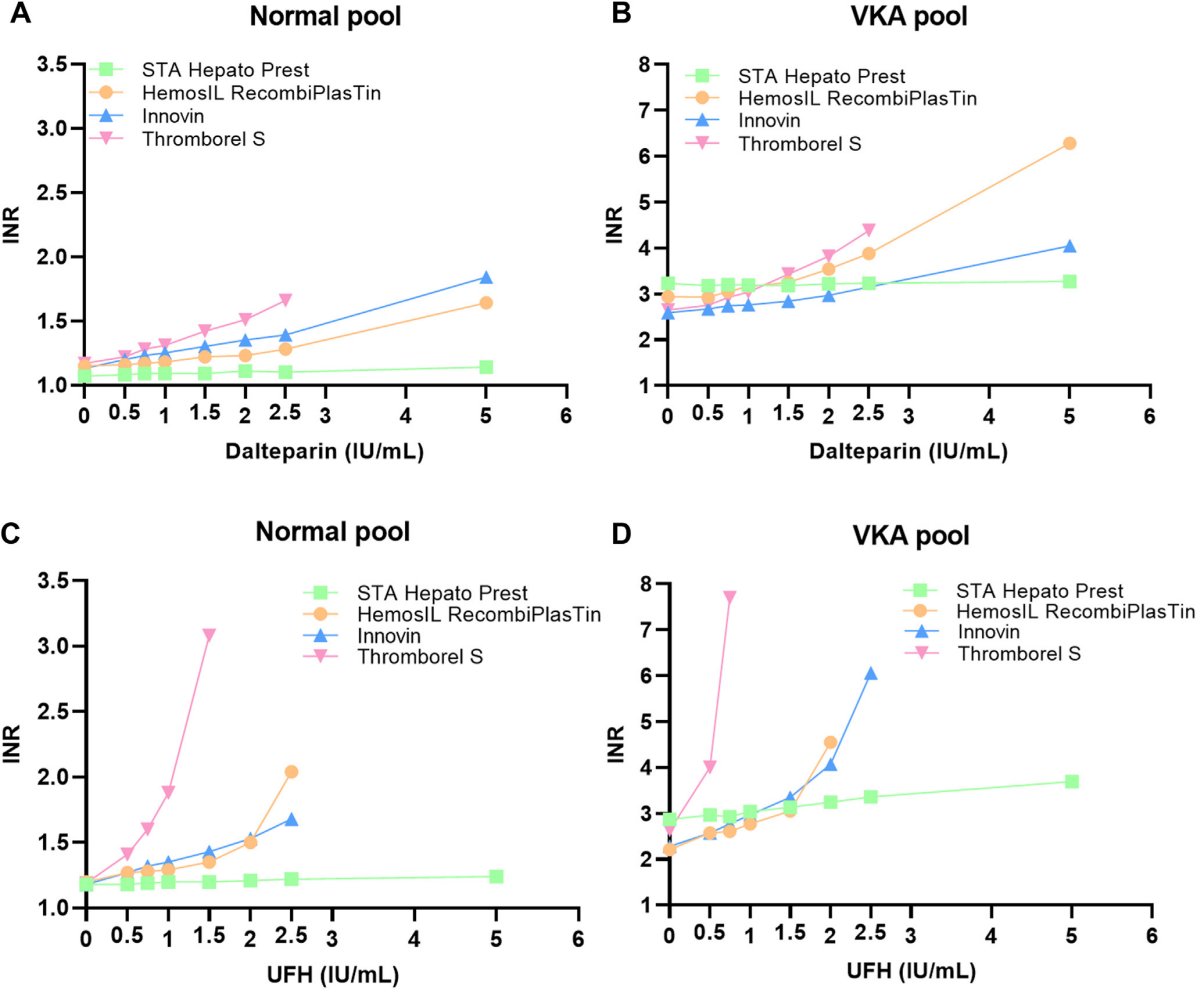
Interference of LMWH or UFH in INR measurement with 4 different coagulation assays. INR was measured with the STA Hepato Prest, HemosIL RecombiPlasTin, Innovin, or Thromborel S assay. Dalteparin (LMWH, A-B) or UFH (C-D) was spiked at various concentrations in pooled plasma of untreated (normal) (A and C) or VKA-treated patients (B and D). INR, international normalized ratio; UFH, unfractionated heparin; VKA, vitamin K antagonist.

The results of the interference testing of heparin in 4 different INR assays are shown in Tables 1 and 2 and Figure 2. Heparin interference was tested using pooled plasma spiked with 7 concentrations of LMWH or UFH and compared with unspiked pooled plasma. Both normal pooled plasma (INR range: 1.07-1.20) and VKA-treated patients’ pooled plasma (INR range: 2.21-3.23) was spiked. The unspiked pools had anti-Xa levels of <0.01, confirming no heparin was present (data not shown). The concentrations of both heparin types in the spiked samples were checked by measuring anti-Xa levels, which were consistent with the expected values of heparin (data not shown). Following the European Federation of Clinical Chemistry and Laboratory Medicine guidelines, the total allowable error of INR is 5.6% [14].

**Table 1:** Interference of LMWH in INR measurement of pooled plasma from untreated and VKA-treated individuals in 4 different coagulation assays.

Dalteparin concentration (IU/mL)	STA Hepato Prest	HemosIL Recombiplastin	Innovin	Thromborel S
Normal				
0.5	0.9%	0.9%	6.2%	4.3%
0.75	1.9%	1.7%	8.8%	9.4%
1	1.9%	2.6%	10.6%	12.0%
1.5	1.9%	6.1%	15.0%	21.4%
2	3.7%	7.0%	19.5%	29.1%
2.5	2.8%	11.3%	23.0%	41.9%
5	6.5%	42.6%	62.8%	x
VKA				
0.5	−1.5%	-0.3%	3.1%	3.8%
0.75	−0.9%	3.7%	5.8%	10.2%
1	−1.2%	7.5%	6.6%	14.7%
1.5	−1.5%	10.5%	9.7%	29.4%
2	−0.3%	20.4%	14.7%	44.2%
2.5	0.0%	32.0%	x	65.3%
5	1.2%	113.6%	56.4%	x

INR was measured using the STA Hepato Prest, HemosIL RecombiPlasTin, Innovin, or Thromborel S assay. Dalteparin (LMWH) was spiked at various concentrations into pooled plasma of normal (untreated) or VKA-treated individuals. Difference in percentage compared to unspiked pooled plasma is shown. x: No value due to unmeasurable high INR.

INR, international normalized ratio; LMWH, low-molecular-weight heparin; VKA, vitamin K antagonist.

**Table 2:** Interference of UFH in INR measurement of pooled plasma from untreated and VKA-treated individuals in 4 different coagulation assays.

UFH concentration (IU/mL)	STA Hepato Prest	HemosIL Recombiplastin	Innovin	Thromborel S
Normal				
0.5	0.0%	5.8%	7.6%	18.5%
0.75	0.8%	6.7%	11.9%	34.5%
1	1.7%	7.5%	14.4%	58.0%
1.5	1.7%	12.5%	21.2%	158.8%
2	2.5%	25.0%	29.7%	x
2.5	3.4%	70.0%	42.4%	x
5	5.1%	x	x	x
VKA				
0.5	3.1%	16.3%	12.7%	52.1%
0.75	2.1%	18.1%	21.5%	192.8%
1	5.9%	25.3%	30.3%	x
1.5	9.1%	38.0%	46.9%	x
2	12.9%	105.9%	78.5%	x
2.5	17.1%	x	165.8%	x
5	28.6%	x	x	x

INR was measured using the STA Hepato Prest, HemosIL RecombiPlasTin, Innovin, or Thromborel S assay. UFH was spiked at various concentrations into pooled plasma of normal (untreated) or VKA-treated individuals. Difference in percentage compared to unspiked pooled plasma is shown. x: No value due to unmeasurable high INR.

INR, international normalized ratio; UFH, unfractionated heparin; VKA, vitamin K antagonist.

The results of LMWH (dalteparin) interference are shown in Table 1 and Figure 2A, B. The STA Hepato Prest assay showed the least interference, with a 6.5% and 1.2% increase in INR at the highest heparin concentration (5 IU/mL) in normal and VKA pooled plasma, respectively. In the HemosIL RecombiPlasTin assay, interference exceeded 5.6% at 0.5 IU/mL in the VKA pool and 1.5 IU/mL in the normal pool. The Dade Innovin assay showed interference >6.2% at 0.5 IU/mL in the normal pool and >7.5% at 1 IU/mL in the VKA pool. The Thromborel S assay had the highest interference, with >9.4% and 10.2% in both pools at 0.75 IU/mL.

UFH, which is commonly used during cardiac surgical procedures but occasionally for anticoagulation bridging, can interfere with INR measurements, despite its short half-life. Therefore, we also tested UFH interference, the results of which are shown in Table 2 and Figure 2C, D. In the STA Hepato Prest assay, UFH caused up to 28.6% interference at 5 IU/mL in the VKA pool but only 5.1% in the normal pool. The HemosIL RecombiPlasTin assay showed >6.1% interference at 1.5 IU/mL in the normal pool and 16.3% at 0.5 IU/mL in the VKA pool. The Dade Innovin assay showed >7.6% interference at 0.5 IU/mL in the normal pool and 12.7% in the VKA pool. The Thromborel S assay had 18.5% interference at 0.5 IU/mL in the normal pool and >52.1% in the VKA pool, with INR undeterminable at concentrations >1 IU/mL. Only STA Hepato Prest could measure INR at 5 IU/mL UFH (Table 2).

In summary, the STA Hepato Prest assay shows no LMWH or UFH interference within the therapeutic range (1.0-2.0 IU/mL) in both VKA and normal plasma. All other assays experience substantial interference, with Thromborel S being the most heparin-sensitive. Heparin interference limits provided by suppliers are: 0.6 IU/mL for Thromborel S, 1.0 IU/mL for HemosIL RecombiPlasTin, and 2.0 IU/mL for both Dade Innovin and STA Hepato Prest. However, the Dade Innovin assay showed interference at levels as low as 0.5 IU/mL LMWH. Higher interference was seen with UFH and VKA-treated pooled plasma. The HemosIL RecombiPlasTin assay was affected by all tested concentrations in plasma pooled from VKA-treated patients, with UFH causing more interference than LMWH. Generally, INR assay accuracy decreases when using plasma from VKA-treated patients, and UFH showed more interference than LMWH at equivalent concentrations, contrary to supplier leaflets.

**Figure 3:**
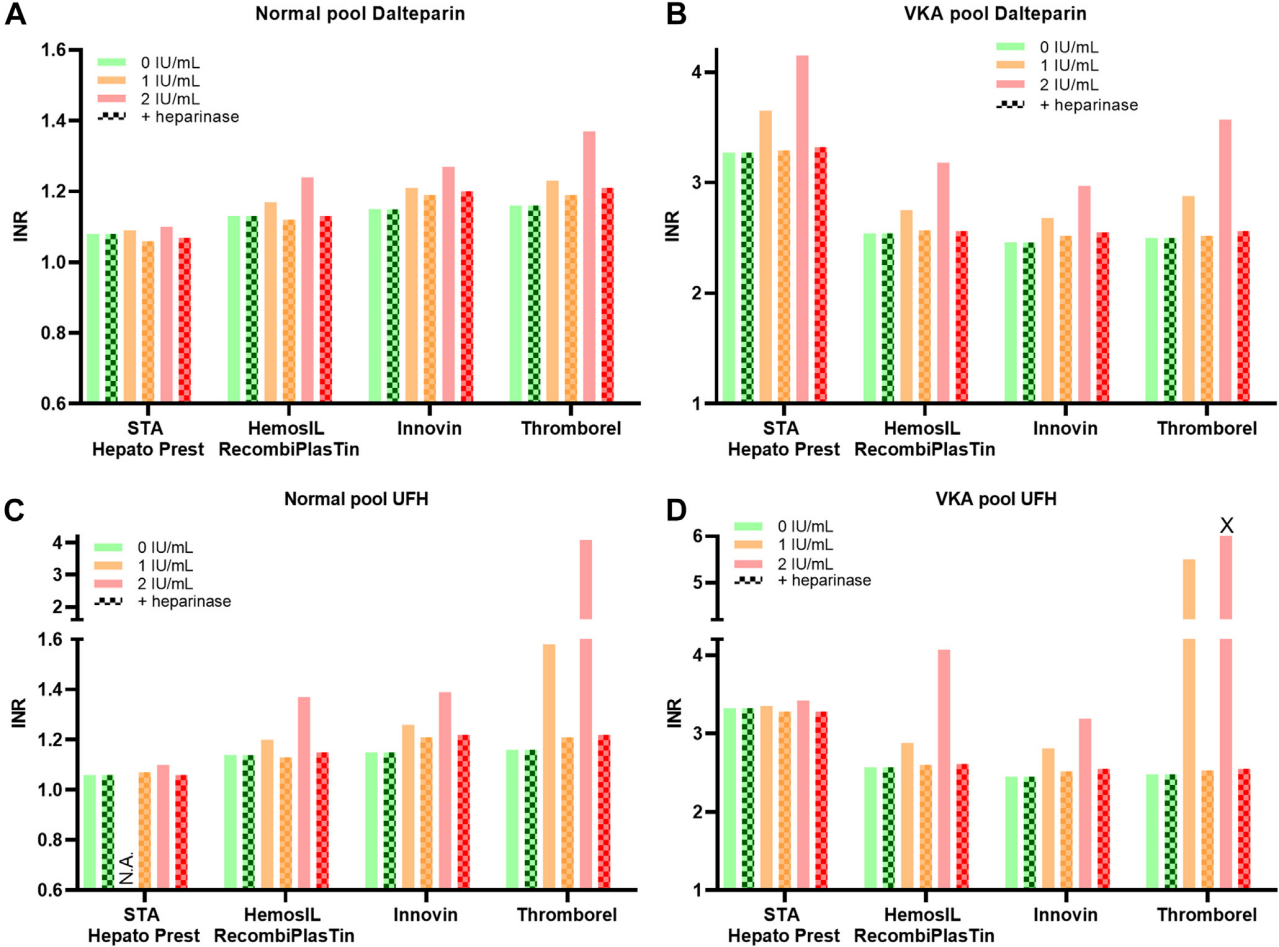
Normalization of INR after addition of heparinase to heparin-spiked plasma pool samples. INR was measured with the STA Hepato Prest, HemosIL RecombiPlasTin, Innovin, or Thromborel S assay. Dalteparin (LMWH, A-B) or UFH (C-D) was spiked at 1.0 IU/mL or 2.0 IU/mL in pooled plasma of untreated (normal) (A and C) or VKA-treated patients (B and D), with and without addition of heparinase (blocked bars). X = INR > 29; N.A. = not analyzed. INR, international normalized ratio; LMWH, low-molecular-weight heparin; UFH, unfractionated heparin; VKA, vitamin K antagonist.

To confirm heparin interference, heparinase was added to samples with 1.0 and 2.0 IU/mL heparin (*in vivo* target concentrations). Anti-Xa measurement confirmed heparin removal (<0.1 IU/mL, data not shown). INR measurements were then normalized to unspiked plasma levels, showing a mean difference of 1.53% for LMWH and 2.11% for UFH compared with unspiked plasma (Figure 3). This experiment confirmed that increased INR levels were due to the presence of heparin in the samples, highlighting that INR accuracy depends on assay-specific heparin interference characteristics.

This study has a few limitations, including the use of only 2 INR assays for postoperative patients on heparin and VKA. These were routine INR measurements; therefore, we did not have any material to measure anti-Xa levels. To enhance the comparison, the *in vitro* experiments included 2 additional commonly used INR assays, with heparin levels confirmed by anti-Xa measurements. Within these *in vitro* experiments, differences in INR were noted even in unspiked pooled plasma, especially VKA pooled plasma (INR range: 2.59-3.23). This was also observed in the External quality Control of diagnostic Assays and Tests program, in which samples are globally distributed and performance of individual laboratories for specific diagnostic assays can be monitored. According to the results of this program, the STA Hepato Prest assay generally measures the highest INR levels. A limitation of the samples that are distributed within the External quality Control of diagnostic Assays and Tests program is that the effect of interferences such as heparin are not evaluated. Even though the STA Hepato Prest has a higher baseline value, using varying spiked heparin concentrations in 2 different INR ranges, as applied in this study, should overcome this bias.

In conclusion, INR measurements should be interpreted cautiously within 72 hours after restarting VKA in postoperative patients receiving LMWH bridging. This caution is due to postoperative coagulation status, the half-lives of vitamin K-dependent factors, and LMWH presence in the plasma. LMWH interference varies across routine coagulation assays, and laboratories should be aware of assay sensitivity to both heparin types, especially when methods are changed, as manufacturers’ heparin limitations may not be accurate for anticoagulated patients. Clinical consequences include risk of overestimation of the anticoagulated state of the patient due to VKA treatment if INR testing is performed before day 4 when assays sensitive to heparin interference are applied, which may result in unjust discontinuation of heparin treatment. Monitoring the anticoagulated state of patients receiving both therapies by specifically measuring anti-Xa levels and INR at trough heparin level may aid in clinical decision making in these cases.
