# Supplementary material for: International normalized ratio measurement during perioperative anticoagulation bridging with low-molecular-weight heparin in patients undergoing heart valve replacement surgery
Source: Res Pract Thromb Haemost. 2024 Nov 5;8(8):102616. doi: 10.1016/j.rpth.2024.102616 (PMC11647484; doi:10.1016/j.rpth.2024.102616)
Supplement: Supplementary file [file mmc1.pdf]

Supplementary Figure 1

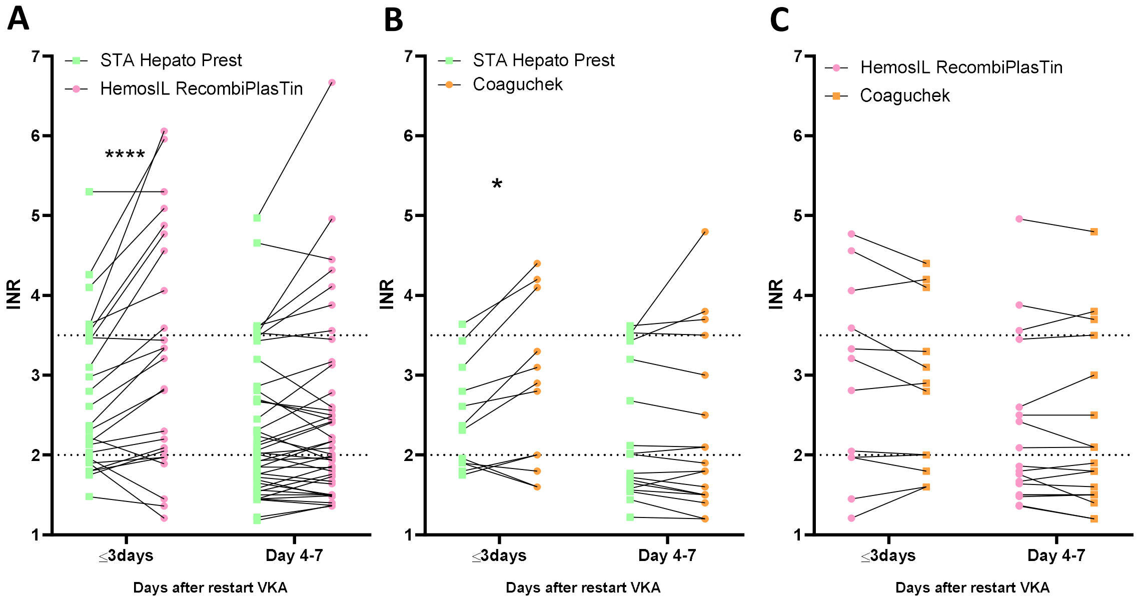

Supplementary figure 1: INR measurement in post-operative anticoagulated patients who received heparin bridging due to valve replacement surgery, including point-of-care testing

(A-C) INR measurement with HemosIL RecombiPlasTin, STA Hepato Prest and Coaguchek within the first 3 days and between day 4-7 after restarting VKA in patients who underwent valve replacement surgery with anticoagulants bridging with LMWH or UFH. Dotted lines represent the therapeutic window of INR. Statistical differences were tested with a two-way ANOVA * =p<0.05, **** p=<0.0001
